# Supplementary material for: A Comparison of the Effect of a 4.4-MHz Radiofrequency Deep Heating Therapy and Ultrasound on Low Back Pain: A Randomized, Double-Blind, Multicenter Trial
Source: J Clin Med. 2022 Aug 26;11(17):5011. doi: 10.3390/jcm11175011 (PMC9457341; doi:10.3390/jcm11175011)
Supplement: Supplementary file 1 [file jcm-11-05011-s001.zip › jcm-1810165-supplementary.pdf]

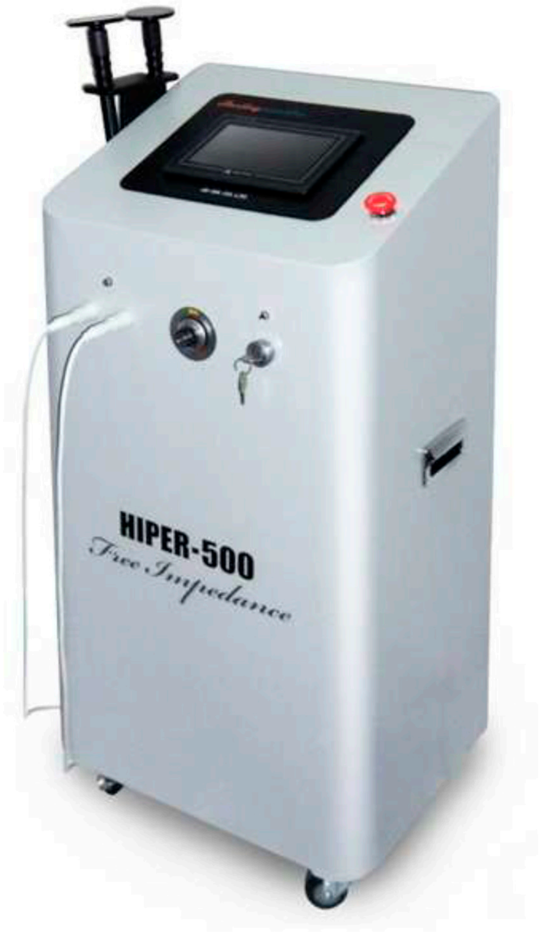

Supplementary Figure S1. HIPER-500 diathermy apparatus® (JS-ON corporation, Seoul, South Korea), two ceramic transducers are connected to the main device.
